# The Omega-3 Fatty Acid Eicosapentaenoic Acid (EPA) Correlates Inversely with Ischemic Brain Infarcts in Patients with Atrial Fibrillation

**DOI:** 10.3390/nu13020651

**Published:** 2021-02-17

**Authors:** Martin F. Reiner, Philipp Baumgartner, Andrea Wiencierz, Michael Coslovsky, Nicole R. Bonetti, Mark G. Filipovic, Giulia Montrasio, Stefanie Aeschbacher, Nicolas Rodondi, Oliver Baretella, Michael Kühne, Giorgio Moschovitis, Pascal Meyre, Leo H. Bonati, Thomas F. Lüscher, Giovanni G. Camici, Stefan Osswald, David Conen, Jürg H. Beer

**Affiliations:** 1 Department of Internal Medicine, Cantonal Hospital of Baden, 5404 Baden, Switzerland; reiner.martin@gmx.ch (M.F.R.); nicole.bonetti@usz.ch (N.R.B.); giulia.montrasio@usz.ch (G.M.); 2 Department of Neurology, University Hospital of Zurich, 8091 Zurich, Switzerland; philipp.baumgartner@usz.ch; 3 Clinical Trial Unit University Hospital of Basel, 4031 Basel, Switzerland; Andrea.Wiencierz@usb.ch (A.W.); michael.coslovsky@usb.ch (M.C.); 4 Center for Molecular Cardiology, Laboratory for Platelet Research, University of Zurich, 8952 Schlieren, Switzerland; tfl@zhh.ch (T.F.L.); giovanni.camici@uzh.ch (G.G.C.); 5 Institute of Anesthesiology, Cantonal Hospital Winterthur, 8400 Winterthur, Switzerland; mark.filipovic@ksw.ch; 6 Department of Cardiology, University Hospital of Basel, 4031 Basel, Switzerland; stefanie.aeschbacher@usb.ch (S.A.); kuehnem@uhbs.ch (M.K.); pascal.meyre@usb.ch (P.M.); stefan.osswald@usb.ch (S.O.); 7 Cardiovascular Research Institute Basel, University Hospital of Basel, 4031 Basel, Switzerland; conend@mcmaster.ca; 8 Department of General Internal Medicine, Bern University Hospital, University of Bern, 3010 Bern, Switzerland; nicolas.rodondi@insel.ch (N.R.); oliver.baretella@insel.ch (O.B.); 9 Institute of Primary Health Care (BIHAM), University of Bern, 3010 Bern, Switzerland; 10 Division of Cardiology, Ospedale Regionale di Lugano, 6900 Ticino, Switzerland; giorgio.moschovitis@eoc.ch; 11 Department of Neurology and Stroke Center, University Hospital Basel, University of Basel, 4031 Basel, Switzerland; leo.bonati@usb.ch; 12 Royal Brompton and Harefield Hospitals, London SW3 6NP, UK; 13 Imperial College, London SW7 2BU, UK; 14 Population Health Research Institute, McMaster University, Hamilton, ON L8S 4L8, Canada

**Keywords:** atrial fibrillation, brain ischemia, eicosapentaenoic acid, omega-3 fatty acids, stroke

## Abstract

The omega-3 fatty acid (n-3 FA) eicosapentaenoic acid (EPA) reduces stroke in patients with atherosclerotic cardiovascular disease. Whether EPA affects stroke or cerebral small vessel dis-ease in patients with atrial fibrillation (AF) remains uncertain. EPA, docosahexaenoic acid (DHA), docosapentaenoic acid (DPA), and alpha-linolenic acid (ALA) were determined by gas chromatography in 1657 AF patients from the Swiss Atrial Fibrillation study. All patients underwent brain MRI to detect ischemic brain infarcts, classified as large noncortical or cortical infarcts (LNCCIs); markers of small vessel disease, classified as small noncortical infarcts (SNCIs), number of microbleeds, and white matter lesion (WML) volumes. Individual and total n-3 FAs (EPA + DHA + DPA + ALA) were correlated with LNCCIs and SNCIs using logistic regression, with numbers of microbleeds using a hurdle model, and WML volumes using linear regression. LNCCIs were detected in 372 patients (22.5%). EPA correlated inversely with the prevalence of LNCCIs (odds ratio [OR] 0.51 per increase of 1 percentage point EPA, 95% confidence interval [CI] 0.29–0.90). DPA correlated with a higher LNCCI prevalence (OR 2.48, 95%CI 1.49–4.13). No associations with LNCCIs were found for DHA, ALA, and total n-3 FAs. Neither individual nor total n-3 FAs correlated with markers of small vessel disease. In conclusion, EPA correlates inversely with the prevalence of ischemic brain infarcts, but not with markers of small vessel disease in patients with AF.

## 1. Introduction

Atrial fibrillation (AF) affects 1% of the global population and increases exponentially with age [1]. It not only augments the risk for ischemic stroke, but also the risk for clinically unrecognized (silent) cerebral infarcts [2,3]. AF is detected in 12% of patients with cerebral ischemia or transient ischemic attack [4]. Anticoagulation is recommended for stroke prevention in AF patients [5,6]; however, despite such treatment, the residual risk for cerebral ischemia remains high [7]. Therefore, additional strategies are needed to reduce the incidence of ischemic brain infarcts in AF.

Omega-3 fatty acids (n-3 FAs) include fish-derived eicosapentaenoic acid (EPA), docosahexaenoic acid (DHA), and docosapentaenoic acid (DPA) as well as plant-derived alpha-linolenic acid (ALA). Both fish- and plant-derived n-3 FAs display anti-thrombotic properties by inhibiting platelet aggregation [8] and blood coagulation [9,10]; in addition, they maintain vascular function by reducing inflammation, atherosclerosis [11,12,13], and endothelial dysfunction [14]. Observational studies have shown that fish-derived, but not plant-derived n-3 FAs correlate inversely with stroke occurrence [15,16,17]. Particularly, supplementation of EPA reduced stroke by 28% in the REDUCE-IT trial (reduction of cardio-vascular events with icosapent ethyl–intervention trial) [18], whereas formulations using both EPA + DHA did not affect stroke outcome in a meta-analysis of randomized controlled trials [19].

However, patients with AF have been underrepresented in clinical trials; therefore, it remains unknown whether n-3 FAs, particularly EPA, also affect stroke in these patients. AF causes thrombogenesis in atrial appendages by promoting platelet activation, coagulation, endothelial dysfunction, and inflammation [20]. The anti-thrombotic, anti-inflammatory, and vascular protective effects of n-3 FAs may prevent thrombogenesis, subsequent embolization, and thus, ischemic brain infarcts as well as cerebral small vessel disease. In this cross-sectional study, we determined n-3 FAs in whole blood of 1657 patients with AF and investigated their association with both symptomatic and clinically unrecognized (silent) ischemic brain lesions as well as small vessel disease, determined by brain MRI. This study was part of the Swiss-AF (Swiss Atrial Fibrillation) study.

## 2. Materials and Methods

### 2.1. Study Population

This cross-sectional study was conducted as part of the Swiss-AF study (ClinicalTri-als.gov Identifier: NCT02105844), an on-going prospective multi-center cohort study that enrolled 2415 patients across 14 centers in Switzerland between 2014 and 2017 with the aim to investigate the prevalence, changes, and underlying mechanisms of structural brain lesions and cognitive decline in AF patients [3,21]. Inclusion criteria were age 65 years or older and a history of documented AF [3,21]. Major exclusion criteria were non-sustained episodes of secondary forms of AF (e.g., after surgery, or severe sepsis), inability to provide informed consent, and acute illness within the last four weeks [3,21]. Of the 2415 patients included in Swiss-AF [3,21], 758 patients were excluded from the current study (727 no brain MRI mainly due to contraindication such as cardiac devices [N= 461] and claustrophobia; 23 had no n-3 FAs measurement; eight missing patient characteristics for statistical adjustments). Finally, 1657 patients were included in this study. In addition, we performed a sensitivity analysis for silent brain infarcts in 1328 patients. In this sensitivity analysis, we excluded patients with previous stroke and transient ischemic attack.

### 2.2. Blood Sampling and Whole Blood Fatty Acid Composition

At baseline, venous EDTA-anticoagulated blood was collected according to standard operating procedures, immediately aliquoted into cryotubes, and stored at −80 °C at a centralized biobank at the University Hospital Basel [21]. Whole blood fatty acids were analyzed as previously described [22]. Samples were transported on dry ice to Omegametrix GmbH, Martinsried, Germany. Samples did not undergo freeze–thaw cycles before analysis of n-3 FAs. Omega-3 FAs were analyzed according to the HS-Omega-3 Index^®^ methodology as previously described [23]. Fatty acid methyl esters were generated from whole blood by acid transesterification and analyzed by gas chromatography using a GC2010 gas chromatograph (Shimadzu, Duisburg, Germany) equipped with a SP2560, 100-m column (Supelco, Bellefonte, PA, USA) with hydrogen as the carrier gas. Fatty acids were identified by comparison with a standard mixture of fatty acids characteristic of erythrocytes. Results are given as fatty acids (EPA, DHA, DPA, ALA, or total n-3 FAs [EPA + DHA + DPA + ALA]) expressed as a percentage of total identified fatty acids after response factor correction. The coefficient of variation for the fatty acid levels was 5%. Analyses were quality controlled according to DIN ISO 15189.

### 2.3. Brain Magnetic Resonance Imaging

Brain MRI was performed at the study baseline. A 1.5-T or 3.0-T scanner was used for brain imaging by applying a 3-dimensional T1-weighted magnetization-prepared rapid gradient echo (MPRAGE) (spatial resolution 1.0 × 1.0 × 1.0 mm^3^), a 2-dimensional axial fluid-attenuated inversion recovery (FLAIR) (spatial resolution 1.0 × 1.0 × 3.0 mm^3^), and 2-dimensional axial diffusion-weighted imaging (spatial resolution 1.0 × 1.0 × 3.0 mm^3^) sequence as well as either a 2-dimensional axial susceptibility-weighted imaging (spatial resolution 1.0 × 1.0 × 3.0 mm^3^) or a 2-dimensional axial T2*-weighted (spatial resolution of 1.0 × 1.0 × 3.0 mm^3^) sequence, as previously described [3]. Brain MRI scans were analyzed centrally in a specialized imaging core laboratory (Medical Image Analysis Centre, Basel, Switzerland) by blinded experts and confirmed by neuroradiologists [3].

### 2.4. End Points

The pre-specified primary endpoint was the prevalence of large noncortical or cortical infarcts (LNCCIs). Large noncortical infarcts were defined by a diameter >20 mm and cortical infarcts were defined as hyperintense lesions on FLAIR involving the cortex irrespective of their size and additional involvement of subcortical areas. [3] LNCCIs are typically attributable to AF. Secondary endpoints were cerebral small vessel disease including prevalence of small noncortical infarcts (SNCIs), number of microbleeds, and white matter lesion (WML) volumes [24]. Small noncortical infarcts were defined as hyperintense lesions on FLAIR, ≤20 mm in diameter on axial sections not involving the cortex, consistent with ischemic infarction in the territory of a perforating arteriole (located in the white matter, internal or external capsule, deep brain nuclei, thalamus, or brainstem), as previously described [3] and according to Wardlaw et al. [24]. FLAIR-hyperintense lesions not meeting the criteria above-mentioned were defined as WMLs. Hyperintense WMLs were additionally graded using the Fazekas scale [25]. Microbleeds were defined as nodular, hypointense lesions on either T2*-weighted or susceptibility-weighted imaging.

### 2.5. Statistical Analysis

We investigated the association of n-3 FAs with the prevalence of LNCCI and SNCIs using logistic regression. We analyzed the association between n-3 FAs with the number of microbleeds using a hurdle model combining a binary regression model for the occurrence of microbleeds with a count data model for all positive numbers of microbleeds. We used truncated negative binomial models as count data models, which describe the association between n-3 FAs and the number of microbleeds among patients with microbleeds. WMLs were detected in 99% of patients; therefore we analyzed the association of n-3 FAs with volumes of WML. The distribution of WML volumes was heavily skewed; therefore, we performed logarithmic transformation prior using a linear regression model. Estimated coefficients were transformed back to the original scale of volumes and represent multiplicative effects. In addition to WML volumes, we analyzed the association between n-3 FAs and the Fazekas score in all patients using ordered logistic regression based on the cumulative link function.

For each endpoint, we analyzed the association with individual n-3 FAs (i.e., EPA, DHA, DPA, or ALA) and total n-3 FAs (EPA + DHA + DPA + ALA). We estimated two models: Model 1 was adjusted for age and sex, and model 2 was adjusted for age, sex, body mass index, smoking status, alcohol consumption, physical activity, coronary artery disease, family history of coronary artery disease, hypertension, diabetes, chronic kidney disease, history of stroke, history of transient ischemic attack, aspirin, anticoagulation, and type of AF.

Finally, we performed a sensitivity analysis for clinically unrecognized (silent) cerebral infarcts in 1328 patients. To this end, we investigated the association of n-3 FAs with LNCCIs in patients without previous stroke or transient ischemic attack. Again, two models were applied. Model 1 was adjusted for age and sex and model 2 was adjusted for age, sex, body mass index, smoking status, alcohol consumption, physical activity, coronary artery disease, family history of coronary artery disease, hypertension, diabetes, chronic kidney disease, aspirin, anticoagulation, and type of AF. Statistical analyses were performed using R Version 4.0.2.

## 3. Results

### 3.1. Study Population

Patients had a mean age of 73 years (standard deviation [SD] 8.4), 27% were female, 13% had a history of stroke, and 26% had a history of coronary artery disease. The majority of patients took anticoagulants (91%) and 14% took aspirin (Table 1). The fatty acid fractions of EPA, DHA, DPA, ALA, and total n-3 FAs were 0.8%, 3.3%, 1.7%, 0.2%, and 6.0% of total identified fatty acids, respectively (Table 2).

### 3.2. Large Cortical and Noncortical Infarcts

LNCCIs were detected in 372 patients (22.5%). We found a significant inverse correlation of EPA with the prevalence of LNCCIs (odds ratio [OR] 0.51 per increase of one percentage point of EPA, 95% confidence interval [CI] 0.29–0.90) (Table 3). A higher prevalence of LNCCIs was detected in patients with higher DPA (OR 2.48, 95% CI 1.49–4.13) (Table 3). Neither DHA (OR 1.10, 95% CI 0.92–1.32), nor ALA (OR 0.88, 95% CI 0.25–3.10) or total n-3 FAs (OR 1.03, 95% CI 0.92–1.15) were significantly associated with the prevalence of LNCCIs (Table 3).

### 3.3. Small Vessel Disease: Small Non-Cortical Infarcts, Number of Microbleeds and White Matter Lesion Volumes

SNCIs were detected in 350 (21.1%) patients. Neither EPA (OR 0.91, 95% CI 0.56–1.49), DHA (OR 1.04, 95% CI 0.87–1.23), DPA (OR 1.19, 95% CI 0.73–1.94), ALA (OR 1.77, 95% CI 0.58–5.38), nor total n-3 FAs (OR 1.03, 95% CI 0.92–1.15) were significantly associated with the prevalence of SNCIs (Table 3).

Microbleeds were present in 365 (22%) patients and the mean (SD) number of microbleeds per patient was 0.5 (2.6). We found no significant correlation of EPA (expB 1.42, 95% CI 0.42–4.77), DHA (expB 0.94, 95% CI 0.68–1.30), DPA (expB 1.24, 95% CI 0.44–3.47), ALA (expB 1.48, 95% CI 0.17–12.79), or total n-3 FAs (expB 1.04, 95% CI 0.81–1.33) with the number of microbleeds (Table 3).

WML were present in 99% of patients. The median WML volume was 3.86 mm3 (interquartile range [IQR] 1.40–9.78). Neither total n-3 FAs (expB 1.03, 95% CI 0.97–1.09) nor EPA (expB 0.99, 95% CI 0.75–1.30), DHA (expB 1.04, 95% CI 0.94–1.14), DPA (expB 1.05, 95% CI 0.80–1.37) or ALA (expB 0.98, 95% CI 0.52–1.85) were significantly associated with WML volumes (Table 3). In addition to WMLs, we analyzed the association of n-3 FAs with the Fazekas score. We found no significant association of individual or total n-3 FAs with the Fazekas score (Supplementary Table S1).

### 3.4. Sensitivity Analysis in Patients with Silent Brain Infarcts

In a sensitivity analysis of patients with silent brain infarcts, patients with a previous stroke or transient ischemic attack were excluded and 1328 patients remained for analysis. LNCCIs were detected in 193 patients (14.5%). EPA correlated with a lower prevalence of LNCCIs (OR 0.48, 95% CI 0.23–1.01), however, the association was not statistically significant (Table 4). Neither DHA (OR 1.01, 95% CI 0.81–1.27), DPA (OR 1.65, 95% CI 0.90–3.06), ALA (OR 1.24, 95% CI 0.27–5.61), nor total n-3 FAs (OR 0.93, 95% CI 0.81–1.07) correlated with the prevalence of LNCCIs (Table 4).

## 4. Discussion

In this cross-sectional study of 1657 patients with AF, we investigated the association of n-3 FAs with ischemic brain infarcts (i.e. LNCCIs) and markers of small vessel disease (i.e. SNCIs, number of microbleeds, and WML volumes). We found that EPA was associated with a lower prevalence of LNCCIs and DPA correlated with a higher LNCCI prevalence. Individual and total n-3 FAs were not associated with markers of small vessel disease such as SNCIs, number of microbleeds, or WML volumes. In a sensitivity analysis including 1328 patients, we investigated the association of n-3 FAs with clinically unrecognized (silent) brain infarcts by excluding patients with previous stroke and transient ischemic attack. We found that EPA correlated non-significantly with the prevalence of LNCCI, thereby supporting our primary analysis. No significant associations were found for DHA, DPA, ALA, and total n-3 FAs.

The findings are in line with our hypothesis that n-3 FAs, particularly EPA, affect ischemic brain infarcts in patients with AF. Previous clinical studies have investigated the association of n-3 FAs with stroke incidence in patients with atherosclerotic cardiovascular disease but not in patients with AF. An observational study in over 50,000 Danish participants determined n-3 FAs in adipose tissue and found that EPA was associated with a lower incidence of strokes caused by large artery atherosclerosis, but not with cardioembolic strokes. [15] Similar to our results, the authors found that DPA correlated with a higher incidence of cardioembolic strokes. [15] Saber et al. analyzed n-3 FAs in plasma and erythrocytes in a subset of patients from three large U.S. cohort studies and found that higher levels of DPA were associated with a lower incidence of cardioembolic strokes, whereas no association of EPA and cardioembolic strokes was found [17]. However, patients with AF were underrepresented and consequently only few cardioembolic strokes were detected in these studies. Moreover, previous studies examined clinically manifest strokes and patients with clinically unrecognized (i.e., silent strokes) were neglected.

In addition to cohort studies, randomized controlled trials examined the effects of n-3 FA supplementation on stroke prevention in patients with atherosclerotic cardiovascular disease, but not in patients with AF. A recent meta-analysis including 13 large randomized controlled trials reported that supplementation using a mixture of EPA and DHA did not reduce the incidence of stroke [19]. Nevertheless, the recently published REDUCE-IT trial found that high doses of EPA (4 g per day) resulted in a marked and significant reduction of fatal or non-fatal strokes by 28% in patients with atherosclerotic cardiovascular disease or a high cardiovascular risk [18]. Although only 5% of the study population had AF [18] and thus, the study was underpowered to evaluate stroke outcome in AF, the results were in line with our findings.

Potential explanations for the observed inverse association between LNCCI prevalence and EPA include its anti-thrombotic and anti-inflammatory properties. Atrial fibrillation causes thrombogenesis, possibly by inducing platelet activation, coagulation, endothelial dysfunction, and inflammation as assessed by higher platelet P-selectin expression, thrombin generation, asymmetric dimethylarginine levels, and platelet-derived soluble CD40 ligand, respectively, in atria of patients in AF [20]. Supplementation of EPA prevented platelet aggregation in response to various agonists [26], reduced the coagulation factors prothrombin and factor V [10] as well as thrombin generation [27], diminished endothelial dysfunction as assessed by flow-mediated dilatation [14], and decreased inflammatory markers including platelet-derived soluble CD40 ligand [28]. Such properties provide plausible mechanisms for the lower prevalence of LNCCIs in AF patients with high levels of EPA.

We found no association of DHA with the prevalence of LNCCI or small vessel dis-ease. Although DHA inhibits platelet aggregation, [29] no effect has been observed on blood coagulation [10], which is considered the predominant mechanism for thrombogenesis in AF. This may in part explain the missing correlation of DHA with ischemic brain lesions in AF.

DPA was associated with a higher prevalence of LNCCIs. This finding is in line with the study by Veno et al., who showed that DPA correlated with cardioembolic strokes, but was in contrast to the study by Saber et al., who reported that DPA was inversely associated with cardioembolic strokes [15]. However, the mechanisms of DPA in thrombus formation have not been evaluated in experimental studies and intervention trials have focused primarily on the supplementation of EPA or EPA plus DHA [19]. Our exploratory analysis of DPA suggests that it might affect ischemic stroke in AF. However, the role of DPA in cardio-embolic strokes remains speculative and our results should be considered as hypothesis generating.

ALA was not associated with the prevalence of LNCCIs or markers of small vessel disease. Previous studies reported that ALA improved endothelial function [30], reduced vascular inflammation [12], and inhibited platelet adhesion and aggregation [31,32]. The well-described anti-inflammatory and anti-thrombotic effects in vitro and in rodents, however, did not translate to significant clinical results. Our observations are in line with a previous meta-analysis of cohort studies showing no significant association of ALA with incidence of stroke [33].

The strengths of our study include (i) the large sample size of 1657 patients with well-classified AF, largely treated with anticoagulants (90.5%); (ii) the evaluation of n-3 FA content in whole blood by a standardized method, which reliably reflects n-3 FA uptake independent of inter-individual bioavailability, and is considered stable up to several years [34]; and (iii) the detection and classification of ischemic brain lesions by brain MRI, which allowed for the identification of brain lesions not only in symptomatic patients but also in patients with silent brain infarcts [3]. Limitations of our study were (i) the cross-sectional design, which does not infer causal relationships; (ii) the study was performed in patients 65 years of age or older in Switzerland and although blood levels of n-3 FAs are comparable to young and healthy adults [35], the findings may not be generalizable to the overall population; (iii) the lack of information on n-3 FA supplementation; and (iv) despite extensive adjustments, it cannot be excluded that our results were due to chance or residual confounding.

In conclusion, EPA was inversely associated with the prevalence of LNCCIs, but not with markers of small vessel disease in patients with AF. In addition, we found that high levels of DPA were associated with a higher prevalence of LNCCIs. Our findings suggest that EPA may not only prevent ischemic stroke in patients with atherosclerotic cardiovascular disease, but may also affect ischemic brain lesions in patients with AF. The relevance of this observation remains to be determined in intervention trials.

## Figures and Tables

**Table 1:** Baseline characteristics. Values were missing for smoking (0.1%), sport (0.1%), family history of coronary artery disease (0.1%), and kidney failure (0.1%).

Overall Population	N = 1657
Mean age (SD)	72.5 (8.4)
Female (%)	453 (27.3)
Median BMI (IQR)	26.9 (24.3, 30.3)
Smoking (%)	
Never	734 (44.3)
Past	800 (48.3)
Active	123 (7.4)
Median alcohol units per day (IQR)	0.6 (0.1, 1.3)
Physical activity (%)	802 (48.4)
Coronary artery disease (%)	425 (25.6)
Hypertension (%)	1136 (68.6)
Diabetes mellitus (%)	251 (15.1)
Kidney failure (%)	294 (17.7)
Previous stroke (%)	221 (13.3)
Previous TIA (%)	145 (8.8)
Family history coronary artery disease (%)	
Yes	610 (36.8)
Unknown	183 (11.0)
Aspirin (%)	234 (14.1)
Anticoagulants (%)	1499 (90.5)
AF type (%)	
Paroxysmal	761 (45.9)
Persistent	495 (29.9)
Permanent	401 (24.2)
Mean CHA_2_DS_2_-VASc score (SD)	3.3 (1.7)

AF = atrial fibrillation, IQR = interquartile range, SD = standard deviation, TIA = transient ischemic attack.

**Table 2:** Omega-3 fatty acid fractions. Total and individual n-3 FAs were determined in whole blood by gas chromatography and are expressed as percentage of total identified fatty acids. Total omega-3 fatty acids include EPA + DHA + DPA + ALA.

Overall Population	Mean Fatty Acid Fraction
N = 1657	% (SD)
Eicosapentaenoic acid (EPA)	0.8 (0.3)
Docosahexaenoic acid (DHA)	3.3 (0.8)
Docosapentaenoic acid (DPA)	1.7 (0.3)
Alpha-linolenic acid (ALA)	0.2 (0.1)
Total Omega-3 fatty acids	6.0 (1.2)

ALA = alpha-linolenic acid, DHA = docosahexaenoic acid, DPA = docosapentaenoic acid, EPA = eicosapentaenoic acid, n-3 FAs = Omega-3 fatty acids.

**Table 3:** Associations of n-3 FAs with clinical endpoints. Model 1 was adjusted for age and sex, model 2 was adjusted for age, sex, body mass index, smoking status, alcohol consumption, physical activity, coronary artery disease, family history of coronary artery disease, hypertension, diabetes, chronic kidney disease, history of stroke, history of transient ischemic attack, aspirin, anticoagulation and type of atrial fibrillation. Total omega-3 fatty acids represent EPA + DHA + DPA + ALA. Data for the prevalence of large noncortical and cortical infarcts as well as small noncortical infarcts are given as odds ratio (95% confidence interval), and data for number of microbleeds and volumes of white matter lesions are given as exponentiation of the beta coefficient (95% confidence interval).

Prevalence of Large Noncortical and Cortical Infarcts (LNCCIs)	Model 1	Model 2
Eicosapentaenoic acid (EPA)	0.50 (0.30–0.83)	0.51 (0.29–0.90)
Docosahexaenoic acid (DHA)	1.15 (0.97–1.35)	1.10 (0.92–1.32)
Docosapentaenoic acid (DPA)	2.68 (1.70–4.21)	2.48 (1.49–4.13)
Alpha-linolenic acid (ALA)	0.73 (0.23–2.31)	0.88 (0.25–3.10)
Total Omega-3 fatty acids	1.06 (0.96–1.17)	1.03 (0.92–1.15)
Prevalence of small		
noncortical infarcts (SNCIs)		
Eicosapentaenoic acid (EPA)	0.92 (0.57–1.47)	0.91 (0.56–1.49)
Docosahexaenoic acid (DHA)	1.05 (0.89–1.24)	1.04 (0.87–1.23)
Docosapentaenoic acid (DPA)	1.24 (0.78–1.98)	1.19 (0.73–1.94)
Alpha-linolenic acid (ALA)	1.41 (0.47–4.22)	1.77 (0.58–5.38)
Total Omega-3 fatty acids	1.05 (0.94–1.16)	1.03 (0.92–1.15)
Number of microbleeds		
Eicosapentaenoic acid (EPA)	1.38 (0.46–4.15)	1.42 (0.42–4.77)
Docosahexaenoic acid (DHA)	0.86 (0.66–1.14)	0.94 (0.68–1.30)
Docosapentaenoic acid (DPA)	0.72 (0.29–1.78)	1.24 (0.44–3.47)
Alpha-linolenic acid (ALA)	5.43 (0.65–45.36)	1.48 (0.17–12.79)
Total Omega-3 fatty acids	0.92 (0.74–1.15)	1.04 (0.81–1.33)
Volumes of white matter		
lesions (WMLs)		
Eicosapentaenoic acid (EPA)	1.02 (0.77–1.33)	0.99 (0.75–1.30)
Docosahexaenoic acid (DHA)	1.02 (0.93–1.12)	1.04 (0.94–1.14)
Docosapentaenoic acid (DPA)	0.98 (0.76–1.27)	1.05 (0.80–1.37)
Alpha-linolenic acid (ALA)	0.89 (0.47–1.67)	0.98 (0.52–1.85)
Total Omega-3 fatty acids	1.01 (0.96–1.07)	1.03 (0.97–1.09)

AF = atrial fibrillation, ALA = alpha-linolenic acid, DHA = docosahexaenoic acid, DPA = docosapentaenoic acid, EPA = eicosapentaenoic acid, n-3 FAs = Omega-3 fatty acids.

**Table 4:** Sensitivity analysis in patients with silent brain infarcts. In this sensitivity of 1328 patients, patients with previous stroke and transient ischemic attack were excluded. Model 1 was adjusted for age and sex and model 2 was adjusted for age, sex, body mass index, smoking status, alcohol consumption, physical activity, coronary artery disease, family history of coronary artery disease, hypertension, diabetes, chronic kidney disease, aspirin, anticoagulation, and type of atrial fibrillation. Total omega-3 fatty acids represent EPA + DHA + DPA + ALA. Data for the prevalence of large noncortical and cortical infarcts are given as odds ratio (95% confidence interval).

Sensitivity Analysis—Prevalence of Large Noncortical and Cortical Infarcts (LNCCIs)	Model 1	Model 2
Eicosapentaenoic acid (EPA)	0.40 (0.19–0.83)	0.48 (0.23–1.01)
Docosahexaenoic acid (DHA)	1.05 (0.84–1.31)	1.01 (0.81–1.27)
Docosapentaenoic acid (DPA)	1.69 (0.93–3.05)	1.65 (0.90–3.06)
Alpha-linolenic acid (ALA)	1.67 (0.39–7.17)	1.24 (0.27–5.61)

AF = atrial fibrillation, ALA = alpha-linolenic acid, DHA = docosahexaenoic acid, DPA = docosapentaenoic acid, EPA = eicosapentaenoic acid.

## Data Availability

The data presented in this study are not publicly available.

## Supplementary Materials

The following are available online at https://www.mdpi.com/2072-6643/13/2/651/s1, Table S1: Association of n-3 FAs with Fazekas score. Model 1 was adjusted for age and sex, model 2 was adjusted for age, sex, body mass index, smoking status, alcohol consumption, physical activity, coronary artery disease, family history of coro-nary artery disease, hypertension, diabetes, chronic kidney disease, history of stroke, history of transient ischemic attack, aspirin, anticoagulation and type of atrial fibrilla-tion. Total omega-3 fatty acids represent EPA + DHA + DPA + ALA. Data are given as odds ratio (95% confidence interval). AF = atrial fibrillation, ALA = alpha-linolenic acid, DHA = docosahexaenoic acid, DPA = docosapentaenoic acid, EPA = eicosapentaenoic acid.

## Abbreviations

AF	atrial fibrillation
ALA	alpha-linolenic acid
DHA	docosahexaenoic acid
DPA	docosapentaenoic acid
EPA	eicosapentaenoic acid
ExpB	exponentiation of the beta coefficient
LNCCIs	large noncortical or cortical infarcts
n-3 FA	omega-3 fatty acids
SNCIs	small noncortical infarcts
Swiss-AF	swiss atrial fibrillation
WMLs	white matter lesions
