# Supplementary material for: The Omega-3 Fatty Acid Eicosapentaenoic Acid (EPA) Correlates Inversely with Ischemic Brain Infarcts in Patients with Atrial Fibrillation
Source: Nutrients. 2021 Feb 17;13(2):651. doi: 10.3390/nu13020651 (PMC7922349; doi:10.3390/nu13020651)
Supplement: Supplementary file 1 [file nutrients-13-00651-s001.pdf]

Supplementary Materials

Table S1. Association of n-3 FAs with Fazekas score. Model 1 was adjusted for age and sex, model 2 was adjusted for age, sex, body mass index, smoking status, alcohol consumption, physical activity, coronary artery disease, family history of coro-nary artery disease, hypertension, diabetes, chronic kidney disease, history of stroke, history of transient ischemic attack, aspirin, anticoagulation and type of atrial fibrilla-tion. Total omega-3 fatty acids represent EPA+DHA+DPA+ALA. Data are given as odds ratio (95% confidence interval). AF = atrial fibrillation, ALA = alpha-linolenic acid, DHA = docosahexaenoic acid, DPA = docosapentaenoic acid, EPA = eicosapentaenoic acid.

Fazekas Score	Model 1	Model 2
Eicosapentaenoic acid (EPA)	1.00 (0.67–1.47)	1.02 (0.68–1.53)
Docosahexaenoic acid (DHA)	0.99 (0.86–1.13)	1.00 (0.87–1.15)
Docosapentaenoic acid (DPA)	0.98 (0.67–1.42)	1.03 (0.70–1.52)
Alpha-linolenic acid (ALA)	1.31 (0.52–3.28)	1.68 (0.65–4.29)
Total Omega-3 fatty acids	0.99 (0.91–1.08)	1.02 (0.93–1.11)

Supplemental List of Swiss-AF Study Investigators

University Hospital Basel and Basel University: Stefanie Aeschbacher, Chloe Au-berson, Steffen Blum, Leo Bonati, Selinda Ceylan, David Conen, Simone Doerpfeld, Marc Girod, Peter Hämmerle, Philipp Krisai, Michael Kühne, Christine Meyer-Zürn, Pascal Meyre, Andreas U. Monsch, Christian Müller, Stefan Osswald, , Philipp Reddiess, Javier Ruperti Repilado, Anne Springer, Fabienne Steiner, Christian Sticherling, Thomas Szucs, Gian Voellmin, Leon Zwimpfer. Principal Investigator: Stefan Osswald; Local Principal Investigator: Michael Kühne.

University Hospital Bern: Faculty: Drahomir Aujesky, Urs Fischer, Juerg Fuhrer, Lau-rent Roten, Simon Jung, Heinrich Mattle; Research fellows: Luise Adam, Carole Elodie Aubert, Martin Feller, Claudio Schneider, Axel Loewe, Elisavet Moutzouri; Study nurses: Tanja Flückiger, Cindy Groen, Damiana Rakovic, Rylana Wenger, Lukas Ehr-sam, Alexandra Nuoffer, Nathalie Schwab. Local Principal Investigator: Nicolas Rodondi.

Stadtspital Triemli Zurich: Christopher Beynon, Roger Dillier, Michèle Deubel-beiss, Franz Eberli, Christine Franzini, Isabel Juchli, Claudia Liedtke, Jacqueline Nadler, Thayze Obst, Noreen Tynan, Xiaoye Schneider, Katrin Studerus, Dominik Weishaupt. Local Principal Investigator: Andreas Müller**.**

Cantonal Hospital Baden: Simone Fontana, Silke Kuest, Karin Scheuch, Denise Hischier, Nicole Bonetti, Alexandra Grau, Jonas Villinger, Eva Laube, Philipp Baumgartner, Mark Filipovic, Marcel Frick, Giulia Montrasio, Stefanie Leuenberger, Franziska Rutz. Local Principal Investigator: Jürg-Hans Beer.

Cardiocentro Lugano: Angelo Auricchio, Adriana Anesini, Cristina Camporini, Giulio Conte, Maria Luce Caputo, Francois Regoli. Local Principal Investigator: Tiziano Moc-cetti.

Cantonal Hospital St. Gallen: Roman Brenner, David Altmann, Michaela Gemperle. Local Principal Investigator: Peter Ammann**.**

Cantonal Hospital Fribourg: Mathieu Firmann, Sandrine Foucras. Local Principal Inves-tigator: Daniel Hayoz.

Cantonal Hospital Lucerne: Benjamin Berte, Virgina Justi, Frauke Kellner-Weldon, Brig-itta Mehmann, , Myriam Roth, Andrea Ruckli-Kaeppeli, Ian Russi, Kai Schmidt, Mabelle Young, Melanie Zbinden. Local Principal Investigator: Richard Kobza**.**

Ente Ospedaliero Cantonale Lugano: Jane Frangi-Kultalahti, Anica Pin, Luisa Vicari Local Principal Investigator: Giorgio Moschovitis**.**

University Hospital Geneva: Georg Ehret, Hervé Gallet, Elise Guillermet, Francois Lazeyras, Karl-Olof Lovblad, Patrick Perret, Philippe Tavel, Cheryl Teres. Local Princi-pal Investigator: Dipen Shah.

University Hospital Lausanne: Nathalie Lauriers, Marie Méan, Sandrine Salzmann. Lo-cal Principal Investigator: Jürg Schläpfer**.**
Bürgerspital Solothurn: Andrea Grêt, Jan Novak, Sandra Vitelli. Local Principal Investi-gator: Frank-Peter Stephan**.**

Ente Ospedaliero Cantonale Bellinzona: Jane Frangi-Kultalahti, Augusto Gallino. Local Principal Investigator: Marcello Di Valentino.
University of Zurich/University Hospital Zurich: Fabienne Witassek, Matthias Schwenkglenks.

Medical Image Analysis Center AG Basel: Jens Würfel (Head), Anna Altermatt, Mi-chael Amann, Petra Huber, Esther Ruberte, Tim Sinnecker, Vanessa Zuber.

Clinical Trial Unit Basel: Michael Coslovsky (Head), Pascal Benkert, Gilles Dutilh, Mil-ica Markovic, Patrick Simon.

Schiller AG Baar: **Ramun Schmi**
